# The Impact of Web-Based Continuing Medical Education Using Patient Simulation on Real-World Treatment Selection in Type 2 Diabetes: Retrospective Case-Control Analysis

**DOI:** 10.2196/48586

**Published:** 2023-08-29

**Authors:** Katie Stringer Lucero, Amy Larkin, Stanislav Zakharkin, Carol Wysham, John Anderson

**Affiliations:** 1 Medscape, LLC New York, NY United States; 2 University of Washington School of Medicine Spokane Spokane, WA United States; 3 MultiCare Rockwood Diabetes & Endocrinology Center Spokane, WA United States; 4 The Frist Clinic Nashville, TN United States

**Keywords:** continuing medical education, virtual patient simulation, real-world evidence, evaluation, outcomes, diabetes education, medical education, type 2 diabetes, web-based learning, web-based education

## Abstract

**Background:**

Despite guidelines recommending the use of glucagon-like peptide-1 receptor agonists (GLP-1 RAs) in certain patients with type 2 diabetes (T2D), they are not being prescribed for many of these patients. Web-based continuing medical education (CME) patient simulations have been used to identify clinicians’ practice gaps and improve clinical decision-making as measured within a simulation, but the impact of this format on real-world treatment has not been researched.

**Objective:**

This study aimed to evaluate the effect of a simulation-based CME intervention on real-world use of GLP-1 RAs by endocrinologists and primary care physicians.

**Methods:**

Two evaluation phases of the CME simulation were conducted: phase I, the CME simulation phase, was a paired, pre-post study of 435 physician learners in the United States; and phase II, the real-world phase, was a retrospective, matched case-control study of 157 of the 435 physicians who had claims data available for the study period.

**Results:**

Phase I CME results showed a 29 percentage point increase in correct decisions from pre- to postfeedback (178/435, 40.9% to 304/435, 69.9%; *P*<.001) in selecting treatment that addresses both glycemic control and cardiovascular event protection. Phase II results showed that 39 of 157 (24.8%) physicians in the intervention group increased use of GLP-1 RAs, compared to 20 of 157 (12.7%) in the comparison group. Being in the intervention group predicted GLP-1 RA use after education (odds ratio 4.49; 95% CI 1.45-13.97; *P*=.001).

**Conclusions:**

A web-based CME simulation focused on secondary prevention of cardiovascular events in a patient with T2D was associated with increased use of evidence-based treatment selection in the real world.

## Introduction

The leading cause of morbidity and mortality in people with type 2 diabetes (T2D) is cardiovascular disease (CVD) [1-7]. Cardioprotective benefits of glucagon-like peptide-1 receptor agonists (GLP-1 RAs) have been confirmed when used in patients with T2D. However, despite current guidelines that strongly recommend the use of GLP-1 RAs in patients with T2D who already have or are at high risk for CVD [8-10], most patients who are eligible for these treatments are not receiving them. Multiple studies indicate that less than 8% of patients with T2D and CVD are receiving a GLP-1 RA [11-17]. Moreover, an analysis of the National Health and Nutrition Examination Survey (NHANES) from 2017 to 2018 found that although one-third of sampled patients with diabetes mellitus were eligible for GLP-1 RAs, in 2018 the use of these agents was limited to only 1 in 100 eligible patients [18].

Therapeutic inertia in diabetes care, generally defined as the failure to initiate or advance therapy when a patient’s glycated hemoglobin A_1c_ (HbA_1c_) is too high (typically >7%), significantly increases the risk of myocardial infarction, heart failure, stroke, and the composite of these 3 cardiovascular (CV) events [19]. Epidemiologic data indicate that for every 20 people with T2D with an HbA_1c_ value 1% above a target of 7%, 1 will experience a microvascular complication within 5 years [20]. Physician factors that contribute to therapeutic inertia may include underestimating the number of patients who are not at target HbA_1c_, lack of knowledge of the efficacy and safety of therapeutic agents, resistance to prescribing new medication, and difficulty in keeping up to date with changing guideline recommendations [20,21].

Importantly, it is suspected that primary care physicians (PCPs) are not fully aware of the benefits of GLP-1 RAs shown in CV outcome trials, because these physicians have fewer opportunities for education on CVD and diabetes than specialists [22]. However, therapeutic inertia is reported to be significant even among specialists [23]. Consequently, there is a clear need for education on CV complications of diabetes and the use of new guideline-based treatment approaches to prevent and treat adverse CV outcomes in patients with type 2 diabetes [24].

Continuing medical education (CME) is an effective tool to close physician practice gaps related to T2D among PCPs and can potentially help improve patient outcomes [25-27]. Patient simulation has been used to identify clinicians’ knowledge gaps and prescribing patterns and improve clinical decision-making [24,28-32], but the impact of web-based, simulation-based CME on real-world treatment selection among clinicians who treat patients with T2D has not been researched. The aim of this study was to investigate the effect of web-based CME simulation on the physician selection of cardioprotective antihyperglycemic treatments in real-world clinical practice.

## Methods

### Study Design

**Figure 1:**
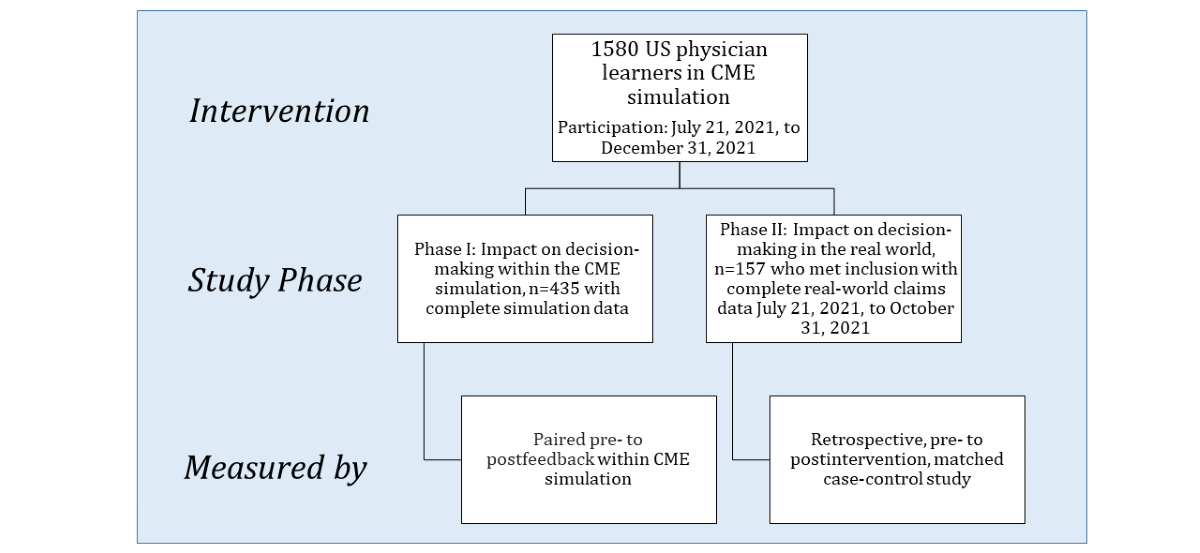
Study design. CME: continuing medical education.

Two study phases were conducted. Phase I focused on decision-making within the MedSims patient simulation, and phase II focused on treatment decisions in the real world (Figure 1).

### Phase I: CME Simulation

A paired, pre-post study was conducted from July 21, 2021, to December 31, 2021. PCPs and endocrinologists who made at least one decision in the simulation and were shown feedback were included.

### Phase II: Real World

A retrospective, matched case-control study was conducted from April 21, 2021, to January 31, 2022. The participation period was July 21, 2021, to October 31, 2021. The participation date upon which the 3 months prior to and after are calculated is herein referred to as the index date. Physicians were included if they had *International Statistical Classification of Diseases and Related Health Problems, Tenth Revision* (*ICD-10*) claims for T2D and prescription data available. Patients were required to have had at least one visit with either a patient simulation CME intervention (intervention group) clinician or a matched control group (comparison group) clinician. In addition, during those visits T2D was coded, and in the 3 months prior to or after the index date an oral T2D treatment was prescribed. Clinician- and patient-level data were obtained through licensed claims data that contains data from over 320 million US patients. A data specialist provided aggregated deidentified clinician-level data to the study lead author (KSL), who analyzed the data.

### Intervention for Phase I and Phase II

The intervention was a web-based MedSims CME simulation activity: Beyond Glycemic Control...Comprehensive Management of T2D [33].

This MedSims patient simulation, which allows learners to order lab tests, make diagnoses, and prescribe treatments in a manner matching the scope and depth of actual practice, contains 2 cases—one focused on primary prevention of CV events in a patient with T2D and one focused on secondary prevention of CV events in a patient with T2D. MedSims simulation provides a customized learning experience with point-of-decision (formative) feedback. Decision-making is open-ended, and opportunities to review decisions allow learners to make real-time changes based on formative feedback. Learners also go through a full review at the end of each case to make adjustments when considering the patient visit as a whole. The intervention addressed the following learning objectives: improve performance associated with ordering appropriate tests to assess glycemic control and CV risk in patients with T2D and improve performance associated with selecting appropriate treatments for primary and secondary prevention of CV events in patients with T2D and CVD.

For the purposes of this study, we limited our investigation to the case that focused on secondary prevention, for which the learners were expected to choose the GLP-1 RA as the most appropriate treatment because GLP-1 RAs are indicated for T2D and secondary CV risk reduction.

### Sample

#### Phase I: CME Simulation

A total of 1580 US physicians (190/1580, 12.03% endocrinologists, 1011/1580 63.99% PCPs) were learners between July 21, 2021, and December 31, 2021. Of those, 435 (74/435, 17% endocrinologists, 287/435, 66% PCPs) made at least one decision, were shown feedback in the secondary prevention case, and were included in this phase of the study.

#### Phase II: Real World

A total of 579 US physicians were learners in the intervention between July 21, 2021, and October 31, 2021. This time frame was chosen because data at the time of pull were available for 3 months of follow-up. Of those physicians, 424 had prescription claims for patients who were on treatment for T2D and had a T2D diagnostic code from July 2020 through January 2022. The 424 physicians who met the inclusion criteria were case-matched with nonparticipant physicians on the following parameters using propensity score matching through Syniti Match [34]: total volume of T2D prescriptions and top 10 prescriptions for patients with T2D (see Multimedia Appendix 1 for prescription codes), profession, specialty, and geographic location (first 2 digits of zip code) using claims data. Of the 424 physicians, 157 intervention-group physicians and their matches had the complete 3 months of preintervention data and 3 months of postintervention data available in licensed claims data. The 157 intervention-group physicians represented 17,004 patients with T2D; 19 of 157 (12.1%) of the physicians were endocrinologists and 108 of 157 (68.8%) were PCPs. The 157 comparison-group physicians represented 16,049 patients with T2D; 16 of 157 (10.2%) of the physicians were endocrinologists and 91 of 157 (58%) were PCPs (Multimedia Appendix 2).

### Measures

#### Phase I and Phase II

##### Participation

For the purposes of this study, learners are defined as those who made a decision and were shown feedback in the intervention.

##### Demographics

Medscape member registration provided country of residence, profession, and specialty.

#### Phase I: CME Simulation

##### Ordering Appropriate Tests to Assess Glycemic Control and CV Risk

Within the CME simulation, learners could order any tests to evaluate the patient with T2D. If they ordered tests that assessed glycemic control and CV risk, they met this learning objective. Appropriate tests to assess glycemic control included HbA_1c_; appropriate tests for CV risk included fasting lipid profile. Learners had the opportunity to choose tests both before and after feedback was given, allowing for revision or reinforcement of their choices.

##### Selection of Appropriate Treatments for Secondary Prevention of CV Events In Patients With T2D

Within the CME simulation, learners could select any available pharmacologic and nonpharmacologic treatment for the simulated patient (or continue the treatment), such as oral glycemic control agents, treatments that are effective for blood glucose management and CV event prevention, weight management therapies, and exercise. If they selected treatments that are effective for CV event prevention and glycemic control, they met this learning objective. Decisions were collected before and after clinical feedback was given.

#### Phase II: Real World

##### Treatment Selection in the Real World

Insurance claims (private, commercial, government) in the United States that indicated a prescription fill were utilized to understand GLP-1 RA use at the clinician and patient levels. GLP-1 RAs included in the analysis were injectable liraglutide, dulaglutide, and semaglutide. Treatments had to have been prescribed for patients with T2D via *ICD-10* codes for T2D and drug names (Multimedia Appendix 1).

### Statistical Analysis

#### Phase I: CME Simulation

Decisions were coded as correct or incorrect pre- and postfeedback. McNemar tests using the Rserve analytics extension for Tableau 2022 were conducted to examine the effect of the CME simulation’s clinical feedback on decision-making to determine whether change in best decisions was statistically significant [35].

#### Phase II: Real World

Logistic regression using SAS (version 9.4; SAS Institute) was conducted to examine the intervention’s impact on clinician GLP-1 RA use for patients with T2D. The dependent variable was GLP-1 RA use postintervention (dummy coded; GLP-1 RA use=0). The independent variable of interest was intervention participation (dummy coded; intervention=1). Controls were chosen because of their association with use of GLP-1 RAs: being a diabetes specialist (dummy coded; endocrinologist=1), prior use of GLP-1 RAs (dummy coded; prior GLP-1 RA use=1), and number of patients with T2D in the 6-month period of the study (discrete).

### Ethical Considerations

According to the US Department of Health and Human Services, this study was exempt from institutional review board approval because it was compliant with the Code of Federal Regulations (CFR) and Medscape privacy policy and leveraged study of existing data that were deidentified to the investigator under 45 CFR 46.104(d)(4); this study qualified for educational research exemption under 45 CFR 46.104(d)(1) [36].

## Results

### Phase I: CME Simulation

Across physicians, there was a 10 percentage point increase in correct responses from pre- to postfeedback (313/435, 71% to 352/435, 80.9%; *P*<.001) for assessing glycemic control and CV risk. There was a 29 percentage point increase in correct responses from pre- to postfeedback (178/435, 40.9% to 304/435, 69.9%; *P*<.001) for selecting treatment that addresses both glycemic control and CV event protection.

### Phase II: Real World

Descriptive results showed that after participation, 69 of 157 (44%) intervention-group clinicians used GLP-1 RAs with their patients with T2D compared with 51 of 157 (32.5%) comparison-group clinicians (Multimedia Appendix 2). Overall, 39 of 157 (24.8%) intervention-group clinicians increased their use of GLP-1 RAs with their patients with T2D compared with 20 of 157 (12.7%) comparison-group clinicians.

Logistic regression results showed that being in the intervention group predicted GLP-1 RA use (odds ratio [OR] 4.49, 95% CI 1.45-13.97; *P*=.001). The intervention group was 4.2 times more likely to use GLP-1 RAs for patients with T2D than the comparison group, controlling for the number of total patients with T2D, being an endocrinologist, and prior use of GLP-1 RAs (Table 1).

**Table 1:** Logistic regression results for use of glucagon-like peptide-1 receptor agonists.

	Estimate	SE	*P* value	Odds ratio (95% CI)
Intercept	–3.89	0.57	<.001	N/A^a^
Total patients with type 2 diabetes	0.005	0.003	.06	1.01 (1.000-1.010)
Intervention	1.50	0.58	.001	4.49 (1.445-13.969)
Glucagon-like peptide-1 receptor agonist use in the preintervention period	5.42	0.65	<.001	225.53 (62.928-808.282)
Endocrinologist	1.09	1.005	.28	2.98 (0.416-21.384)

^a^N/A: not applicable.

## Discussion

### Principal Findings

Results from decisions made within the CME simulation show an improvement in assessment of CV risk and glycemic control and selection of GLP-1 RAs within the simulation, and the matched case-control study shows that participation in the CME simulation was associated with significant increases in use of GLP-1 RAs.

Previous research has shown that short, case-based, web-based CME activities improved knowledge, competence, and self-reported performance in T2D management among health care professionals (HCPs) [37]. The results of this study go beyond the limitation of HCPs’ self-reported performance to suggest that patient simulation CME is reflective of real-world practice behavior, as there was concordance (within the same phase I time frame) in decision-making between the percentage of clinicians who selected GLP-1 RAs in the CME simulation prior to feedback (178/435, 41%) and the entire population of clinicians using GLP-1 RAs for patients with T2D in the real world (394,133/947,437, 40.6%) prior to the intervention. However, there was not concordance between the percentage of clinicians who selected GLP-1 RAs in the CME simulation after feedback (304/435, 69.9%) and the percentage of clinicians using GLP-1 RAs in patients with T2D in the real world who also participated in the CME simulation (69/157, 44%). This difference is likely due to barriers faced in the real world that may limit prescribing (such as insurance coverage and patient readiness for injectables), as well as the limitation inherent in presenting only 1 patient type in the simulation, whereas many different patient types and possible care scenarios exist in the real world. For example, real-world patients may refuse certain treatments, but this possibility was not a factor in the CME simulation; thus, clinicians who participated in the simulation may need additional education that presents them with several patient types.

To provide context for physicians’ use of GLP-1 RAs postintervention, it is helpful to note the reasons they offered within the CME simulation for selecting a treatment for secondary prevention of CV events. The top 6 reasons given by endocrinologists were drug efficacy (25/53, 47%), clinical trials supporting drug use (23/53, 43%), patient profile (20/53, 38%), guideline recommendation (17/52, 33%), familiarity with use (17/52, 33%), and indication for primary and secondary prevention (3/55, 5%). The top 6 reasons given by PCPs were guideline recommendation (80/195, 41%), clinical trials supporting drug use (76/195, 39%), patient profile (72/190, 37.8%), indication for primary and secondary prevention (70/195, 35.9%), efficacy (61/190, 32.1%), and familiarity with use (10/195, 5.1%).

Notably, drug efficacy held nearly inverse positions for endocrinologists and PCPs as a reason for treatment selection. This may indicate that endocrinologists take a more granular view of drug-specific factors described in guidelines [38], which show high efficacy for GLP-1 RAs but only intermediate efficacy for sodium-glucose cotransporter-2 (SGLT2) inhibitors. As nonspecialists, PCPs may be more reliant on guideline decision trees [38], which present the selection of GLP-1 RAs and SGLT2 inhibitors as an “either/or” treatment choice for patients with or at high risk for atherosclerotic CVD (ASCVD). Indeed, only 10 of 195 (5.1%) PCPs who participated in the CME simulation chose familiarity with use as a reason for treatment selection.

The most common reasons offered by endocrinologists for not selecting a treatment for secondary prevention of CV events were being unfamiliar with use (9/20, 45%), the drug being unavailable on the formulary (8/22, 36%), the patient not needing secondary prevention (4/23, 18%), and drug cost (4/23, 18%). The most common reasons given by PCPs were cost (36/92, 39%), being unfamiliar with use (36/92, 36%), the patient being uncomfortable with injection (21/90, 23%), and the drug being unavailable on the formulary (18/91, 20%).

Although in the real world physicians were using GLP-1 RAs with patients with T2D, the majority of their patients with CV event risk factors were still not receiving treatment; only 205/4372 (4.68%) of patients with T2D and ASCVD received GLP-1 RAs in the CME group after the intervention. More education is needed to address barriers to use of this class of drugs with patients who would benefit. Our results suggest that education for endocrinologists should emphasize familiarity with the use of GLP-1 RAs and recognition of patients who would benefit; for PCPs, education should aim to improve familiarity with use and comfort with injection.

### Limitations

Possible limitations of our study are the inability to determine clinicians’ rationale for selecting GLP-1 RAs in practice, lack of randomization, small sample size for non-PCP participants, inability to determine if increased real-world use of GLP-1 RAs could be associated with all patient simulation CME interventions (ie, our results are localized to this intervention), and a follow-up limited to a 3-month period. The 3-month follow-up limits our ability to evaluate whether increased and new prescribing were a durable result associated with the intervention. However, durability of results for this type of study typically wanes the further out from the intervention period the results are measured. Unmeasured confounders such as motivation to take the intervention for the intervention group versus not take it for the control group were not considered. Simply the motivation to undertake the CME simulation could contribute to the effect found. Finally, the study design helped minimize the sampling bias, but there still may be unmeasured confounders. These may include consumption of other content, such as other CME activities, published studies, and collegial conversations, as well as level of motivation to adjust treatment selection.

### Conclusions

A strength of this study is that claims data indicated that before the intervention, the percentage of physicians in the intervention group who treat patients with T2D with GLP-1 RAs (59/157, 37.6%) and the percentage of the total population of US physicians who treat patients with T2D with GLP-1 RAs were comparable (394,133/947,437, 40.6%). In addition, claims data showed that before the intervention, the percentage of patients treated with GLP-1 RAs by the intervention-group physicians (1056/17,004, 6.21%) and the percentage of patients treated with GLP-1 RAs in the US population were also comparable (848,607/13,731,508, 6.18%). These similarities between groups indicate that our study has direct implications for impact on public health. Ultimately, the results are generalizable for clinicians who treat patients with T2D, are members of clinical news and medical education platforms, and engage in web-based, simulation-based CME on the topic of newer T2D treatments that have potential benefits for glycemic control and CV protection.

A case-based virtual patient simulation CME intervention focused on secondary prevention of CV events in a patient with T2D was associated with increased selection of cardioprotective antihyperglycemic treatments in clinical practice among both endocrinologists and PCPs.

## Appendix

Multimedia Appendix 1 Codes used for the study.

Multimedia Appendix 2 Intervention and comparison descriptive statistics.

## Abbreviations

ASCVD: atherosclerotic cardiovascular disease
CFR: Code of Federal Regulations
CME: continuing medical education
CV: cardiovascular
CVD: cardiovascular disease
GLP-1 RA: glucagon-like peptide-1 receptor agonist
HbA1c: glycated hemoglobin A1c
*ICD-10*: *International Statistical Classification of Diseases and Related Health Problems, Tenth Revision*
NHANES: National Health and Nutrition Examination Survey
PCP: primary care physician
SGLT2: sodium-glucose cotransporter-2
T2D: type 2 diabetes

## Data Availability

The data sets generated and/or analyzed during this study are not publicly available due to Medscape member privacy. Access to the data is restricted to employees of Medscape who have permission. Statistical code and study protocol are available upon request to the corresponding author.
