# Supplementary material for: The Impact of Web-Based Continuing Medical Education Using Patient Simulation on Real-World Treatment Selection in Type 2 Diabetes: Retrospective Case-Control Analysis
Source: JMIR Med Educ. 2023 Aug 29;9:e48586. doi: 10.2196/48586 (PMC10498312; doi:10.2196/48586)
Supplement: Multimedia Appendix 1 [file mededu_v9i1e48586_app1.docx]

Multimedia Appendix 1: Codes used for the study.

### National Drug Codes (NDCs) used for propensity score matching based on glucagon-like peptide-1 receptor agonist use at baseline and top 10 treatments for patients with type 2 diabetes

NDC:00054474125, NDC:00054474225, NDC:00527293341, NDC:00603533831, NDC:70954005820, NDC:00143147701, NDC:00054472825, NDC:00603533928, NDC:00378064010, NDC:00054981725, NDC:59746017206, NDC:00591544201, NDC:00603533732, NDC:00527293441, NDC:00603533828, NDC:00603533821, NDC:00603533715, NDC:00054001820, NDC:00143973810, NDC:00143973801, NDC:00603533921, NDC:00591505210, NDC:00143974010, NDC:70954005920, NDC:59746017210, NDC:00603533932, NDC:00591544310, NDC:00603533832, NDC:00378064205, NDC:00603533815, NDC:00054472831, NDC:00591544301, NDC:00591544210, NDC:70954006020, NDC:00054001729, NDC:00378064110, NDC:00143973910, NDC:00054001725, NDC:00591544305, NDC:59746017306, NDC:59746017310, NDC:59746017509, NDC:00054001925, NDC:00054001829, NDC:00143973805, NDC:59746017506, NDC:00054001825, NDC:65862020299, NDC:65862020399, NDC:65862020390, NDC:65862020290, NDC:31722070290, NDC:31722070210, NDC:65862020190, NDC:31722070110, NDC:68382013616, NDC:65862020199, NDC:31722070090, NDC:43547036109, NDC:00781572001, NDC:68382013716, NDC:54838055550, NDC:23155054842, NDC:23155054831, NDC:62756035664, NDC:45963053930, NDC:16714020030, NDC:57237007730, NDC:00054006447, NDC:70860077602, NDC:23155054731, NDC:00409475518, NDC:68001024617, NDC:36000001225, NDC:00641608001, NDC:00378773493, NDC:00093023356, NDC:23155054742, NDC:65162069179, NDC:57237007630, NDC:63304045830, NDC:00641608025, NDC:68462015813, NDC:00781523964, NDC:55150012502, NDC:60505613000, NDC:65862018830, NDC:60505613005, NDC:62756024064, NDC:00641607801, NDC:68462010630, NDC:45963053830, NDC:55111015430, NDC:65862039110, NDC:00641607825, NDC:57237007810, NDC:00409475503, NDC:57237007530, NDC:00378773293, NDC:00781523864, NDC:65862018730, NDC:68462010530, NDC:55111015330, NDC:68462015713, NDC:57237007710, NDC:65862039010, NDC:00006057761, NDC:53746017810, NDC:23155010201, NDC:65862000805, NDC:57664039758, NDC:53746021805, NDC:68645054459, NDC:65862001099, NDC:00093726710, NDC:68645058459, NDC:70010006510, NDC:67877041305, NDC:67877056310, NDC:62756014201, NDC:68382002810, NDC:70010049101, NDC:65862001001, NDC:68645053959, NDC:00378718705, NDC:57664039753, NDC:68645054559, NDC:53746017805, NDC:00093104810, NDC:67877056105, NDC:70010006310, NDC:67877015905, NDC:68645058259, NDC:51224000760, NDC:70010049105, NDC:70010049110, NDC:00378718505, NDC:67877056110, NDC:65862000801, NDC:23155010405, NDC:53746017801, NDC:68382076010, NDC:23155010205, NDC:65862001005, NDC:62756014202, NDC:23155010410, NDC:23155010210, NDC:68382075810, NDC:65862000899, NDC:00185062001, NDC:00591040810, NDC:00185010110, NDC:00591040801, NDC:43547035210, NDC:00185010101, NDC:43547041611, NDC:00591040701, NDC:68645055754, NDC:43547041711, NDC:00185061010, NDC:43547035610, NDC:00185061001, NDC:00185062010, NDC:68180051501, NDC:68645055554, NDC:68180051401, NDC:00603421232, NDC:00603421132, NDC:54458099710, NDC:54458099610, NDC:43547035310, NDC:43547035410, NDC:68180098001, NDC:68180098101, NDC:68645055154, NDC:68180051701, NDC:68180051301, NDC:68180051802, NDC:68180051201, NDC:68180052002, NDC:43547035211, NDC:68180097903, NDC:68180051801, NDC:68180052001, NDC:43547035611, NDC:68180051902, NDC:68180051901, NDC:68645055354, NDC:68180051202, NDC:68645055254, NDC:68180051503, NDC:43547035311, NDC:43547035411, NDC:68180051703, NDC:68180051403, NDC:68180051303, NDC:68180098103, NDC:68180098003, NDC:13107002001, NDC:31722094101, NDC:00406012310, NDC:57664017688, NDC:53746011005, NDC:00121077216, NDC:00591320201, NDC:53746011001, NDC:00603389132, NDC:27808003502, NDC:43386035801, NDC:00603388728, NDC:00591260505, NDC:00603388721, NDC:64376064801, NDC:00406012505, NDC:00591261205, NDC:00603389032, NDC:00603388732, NDC:00603388128, NDC:00603389128, NDC:00406012405, NDC:27808003503, NDC:00591034905, NDC:00406036501, NDC:53746010905, NDC:31722099601, NDC:43386035701, NDC:00591217205, NDC:00406036505, NDC:00603389121, NDC:00591320205, NDC:00603389028, NDC:00406035705, NDC:43386035601, NDC:00406012501, NDC:00603389021, NDC:00406012401, NDC:00406012305, NDC:53746010901, NDC:00406012301, NDC:59746038210, NDC:16729018301, NDC:68645055754, NDC:57237000299, NDC:00591034705, NDC:50228014610, NDC:29300013005, NDC:23155004710, NDC:00228282011, NDC:16729018217, NDC:68180051802, NDC:00378081005, NDC:68180052002, NDC:68180051801, NDC:68180052001, NDC:68645051054, NDC:68180051902, NDC:68180051901, NDC:23155000810, NDC:00603385632, NDC:00172208380, NDC:16729018201, NDC:29300012810, NDC:16729018317, NDC:68180063709, NDC:55111012105, NDC:00378395005, NDC:68645045954, NDC:68645048054, NDC:00378395105, NDC:55111012190, NDC:63304082990, NDC:63304082890, NDC:59762015701, NDC:63304082905, NDC:59762015501, NDC:70377002711, NDC:63304082805, NDC:68645046054, NDC:68382024916, NDC:16729004417, NDC:00378395377, NDC:00378395205, NDC:55111012390, NDC:16729004617, NDC:68645048154, NDC:55111012290, NDC:00093505698, NDC:59762015702, NDC:68645056854, NDC:59762015601, NDC:55111012205, NDC:59762015602, NDC:68382025116, NDC:55111012305, NDC:59762015502, NDC:68382025016, NDC:60505267108, NDC:70377002811, NDC:70377002911, NDC:16729004517, NDC:00093505998, NDC:00093505898, NDC:60505267109, NDC:00378395077, NDC:00378395277, NDC:00378395177, NDC:60505257808, NDC:60505257809, NDC:60505257908, NDC:60505258008, NDC:60505258009, NDC:60505257909, NDC:50090513900, NDC:50090605100, NDC:00169477290, NDC:00169477297, NDC:00169413290, NDC:50090513800, NDC:00169431401, NDC:70518214300, NDC:00169430701, NDC:00169430301, NDC:00169413297, NDC:00169430390, NDC:00169430393, NDC:50090594900, NDC:00169477211, NDC:00169413001, NDC:00169413611, NDC:00169413211, NDC:00169431413, NDC:00169431430, NDC:00169450514, NDC:00169477212, NDC:00169430330, NDC:00169430730, NDC:00169430313, NDC:00169430713, NDC:00169413602, NDC:00169413013, NDC:00169413212, NDC:00002318261, NDC:50090546700, NDC:54568043463, NDC:54568043371, NDC:50090348400, NDC:54568043471, NDC:54568043363, NDC:00002223661, NDC:00002143461, NDC:50090348300, NDC:00002143361, NDC:00002318201, NDC:00002223601, NDC:00002143401, NDC:00002143301, NDC:00002318280, NDC:00002223680, NDC:00002143480, NDC:00002143380, NDC:00169291190, NDC:50090450300, NDC:00169291197, NDC:00169280090, NDC:00169280097, NDC:00169280013, NDC:00169406098, NDC:00169406097, NDC:00169406090, NDC:50090425700, NDC:00169406099, NDC:50090285300, NDC:54569650700, NDC:00169291115, NDC:00169280015, NDC:00169406012, NDC:00169406013

### List of the *International Statistical Classification of Diseases and Related Health Problems, Tenth Revision* (*ICD-10*) codes for patient inclusion

E1100, E1101, E1121 E1122, E1129, E11311, E11319, E11321, E11329, E11331, E11339, E11341, E11349, E11351, E11359, E1136, E1139, E1140, E1141, E1142, E1143, E1144, E1149, E1151, E1152, E1159, E11610, E11618, E11620, E11621, E11622, E11628, E11630, E11638, E11641, E11649, E1165, E1169, E118, E119

### Glucagon-like peptide-1 receptor agonists as dependent variable for logistic regression liraglutide, dulaglutide, semaglutide
