# Supplementary material for: The Impact of Web-Based Continuing Medical Education Using Patient Simulation on Real-World Treatment Selection in Type 2 Diabetes: Retrospective Case-Control Analysis
Source: JMIR Med Educ. 2023 Aug 29;9:e48586. doi: 10.2196/48586 (PMC10498312; doi:10.2196/48586)
Supplement: Multimedia Appendix 2 [file mededu_v9i1e48586_app2.docx]

Multimedia Appendix 2: Intervention and comparison descriptive statistics.

Table 1. Intervention and Comparison Descriptive Statistics (Phase II)

|  | Intervention group n = 157 | Comparison group n = 157 | Between-group difference *P* value^a^ |
| --- | --- | --- | --- |
| Endocrinologists | 12.1% | 10.1% | .51 |
| Primary care physicians^b^ | 70.1% | 62.4% | .03 |
| Other specialists | 17.2% | 27.4% | .15 |
| Mean total selected prescriptions^c^ | 246.7 | 252.1 | .92 |
| Mean total patients with type 2 diabetes (T2D) per physician in 6-month period | 108.3 | 102.2 | .73 |
| Mean number of patients on a glucagon-like peptide-1 receptor agonist (GLP-1 RA) pre-intervention period | 6.7 | 5.6 | .63 |
| Mean number of patients on a GLP-1 RA post-intervention period | 7.2 | 5.5 | .51 |
| % of clinicians who selected GLP-1 RAs pre-intervention period | 37.6% | 33.1% | .48 |
| % of clinicians who selected GLP-1 RAs post-intervention period | 44.0% | 32.4% | .037 |
| % of clinicians who increased GLP-1 RA selection and were prior users | 17.8% | 10.8% | .049 |
| % of clinicians who selected GLP-1 RAs for the first time | 7.0% | 1.9% | .029 |
| % of clinicians who had flat or reduced selection of GLP-1 RAs | 19.1% | 19.8% | .58 |
| % of clinicians who did not select GLP-1 RAs for patients with T2D in the post-intervention period | 55.4% | 67.5% | .04 |

^a^Note: Between-group mean differences were assessed using independent samples t-tests; between-group proportion differences were assessed using chi-square tests

^b^Includes obstetricians/gynecologists and pediatricians

^c^Includes GLP-1 RAs and top 10 prescriptions used by patients with T2D (full list in Multimedia Appendix 2. Codes used for the study.)
